# D-dimer and CoV-2 spike-immune complexes contribute to the production of PGE2 and proinflammatory cytokines in monocytes

**DOI:** 10.1371/journal.ppat.1010468

**Published:** 2022-04-06

**Authors:** Yun-Jong Park, David Acosta, Russell Vassell, Juanjie Tang, Surender Khurana, Carol D. Weiss, Hana Golding, Marina Zaitseva

**Affiliations:** Division of Viral Products, Center for Biologics Evaluation and Research (CBER), Food and Drug Administration (FDA), Silver Spring, Maryland, United States of America; University of Alabama at Birmingham, UNITED STATES

## Abstract

An overreactive inflammatory response and coagulopathy are observed in patients with severe form of COVID-19. Since increased levels of D-dimer (DD) are associated with coagulopathy in COVID-19, we explored whether DD contributes to the aberrant cytokine responses. Here we show that treatment of healthy human monocytes with DD induced a dose dependent increase in production of pyrogenic mediator, Prostaglandin E2 (PGE2) and inflammatory cytokines, IL-6 and IL-8. The DD-induced PGE2 and inflammatory cytokines were enhanced significantly by co-treatment with immune complexes (IC) of SARS CoV-2 recombinant S protein or of pseudovirus containing SARS CoV-2 S protein (PVCoV-2) coated with spike-specific chimeric monoclonal antibody (MAb) containing mouse variable and human Fc regions. The production of PGE2 and cytokines in monocytes activated with DD and ICs was sensitive to the inhibitors of β2 integrin and FcγRIIa, and to the inhibitors of calcium signaling, Mitogen-Activated Protein Kinase (MAPK) pathway, and tyrosine-protein kinase. Importantly, strong increase in PGE2 and in IL-6/IL-8/IL-1β cytokines was observed in monocytes activated with DD in the presence of IC of PVCoV-2 coated with plasma from hospitalized COVID-19 patients but not from healthy donors. The IC of PVCoV-2 with convalescent plasma induced much lower levels of PGE2 and cytokines compared with plasma from hospitalized COVID-19 patients. PGE2 and IL-6/IL-8 cytokines produced in monocytes activated with plasma-containing IC, correlated well with the levels of spike binding antibodies and not with neutralizing antibody titers. Our study suggests that a combination of high levels of DD and high titers of spike-binding antibodies that can form IC with SARS CoV-2 viral particles might accelerate the inflammatory status of lung infiltrating monocytes leading to increased lung pathology in patients with severe form of COVID-19.

## Author summary

The pathology of severe COVID-19 is associated with massive inflammation and activation of coagulation systems leading to thrombosis and disseminated intravascular coagulation. D-dimer, a degradation product of fibrinogen, accumulates in the blood when thrombus is dissolved. D-dimer is increased following coagulation activation and high levels of D-dimer correlate with poor prognosis in patients with severe disease. Advanced stages of COVID-19 are also associated with high viral loads and presence of Immune Complexes, i. e. viral particles coated with anti-spike protein antibodies. Here we investigated a link between elevated levels of D-dimer, immune complexes, and inflammation in COVID-19 using human monocytes. Our data showed that D-dimer alone induced prostaglandin E2 (PGE2), a final trigger of fever, and inflammatory cytokines, IL-6/IL-8/IL-1β in healthy monocytes. Importantly, PGE2 and cytokines produced by monocytes were significantly increased when monocytes were incubated with D-dimer and immune complexes of SARS CoV-2 viral particles coated with antibodies from COVID-19 patients. These data showed that D-dimer and immune complexes co-amplify the inflammatory responses of monocytes. Understanding the relationship between coagulation cascade and inflammatory response in severe COVID-19 is critical for designing therapies and treatments to improve outcomes of the disease.

## Introduction

Coronavirus disease 2019 (COVID-19), an acute respiratory tract infection that emerged in late 2019, is caused by a novel coronavirus, severe acute respiratory syndrome coronavirus 2 (SARS-CoV-2) [1–3]. Although most patients experience mild to moderate disease, 5 to 10% progress to critical pneumonia and acute respiratory failure [4–6]. The high morbidity and mortality of COVID-19 is associated with dysregulated immune responses as evidenced by the presence of high levels of inflammatory markers including C-reactive protein, inflammatory cytokines and chemokines, and Prostaglandin E2 (PGE2) in the circulation [6–11]. The hyperactive immunopathology is postulated as a major cause of morbidity and mortality in COVID-19, however, the mechanisms of uncontrolled inflammatory responses underlying the pathogenesis of the disease remain largely unknown.

The evidence that monocytes and macrophages play a critical role in the lung inflammation and in the overall pathophysiology of severe COVID-19 is rapidly accumulating [12]. Immune scoring of COVID-19 lung biopsies revealed massive myeloid infiltration, specifically by monocytes, M1 macrophages, and neutrophils [13]. Single-cell RNA sequencing analysis (scRNA seq) of Bronchoalveolar fluid (BALF) showed increase in the proportion of monocytes/macrophages in BALF up to 80% in patients with severe COVID-19 compared with 60% and 40% in patients with mild diseases and healthy subjects, respectively [14]. In addition, BALF from patients with severe disease contain increased levels of CCL2 and CCL7 chemokines known to recruit CCR2+ monocytes to the inflamed lungs [15]. Whether monocytes and macrophages are targets of SARS-CoV-2 in vivo is still under debate. The SARS CoV-2 receptor, the ACE2 protein, was not found on most tissue resident macrophages by scRNA [16]. Monocytes and monocyte-derived macrophages were shown to sustain only abortive but not productive infection with SARS-CoV-2 in vitro [17], suggesting that proinflammatory activity of monocytes and macrophages in patients with severe disease may be triggered by pathways unrelated to productive infection.

Monocytes express various Fc gamma-receptors (FcγR) that bind the constant region of IgG and that are primarily involved in recognition of IgG-opsonized pathogens leading to clearance of the pathogen but that can also participate in immune complex (IC)-mediated inflammation. Monocytes express both activating FcγR (FcγRI, FcγRIIa, and FcγRIIIa) that trigger production of proinflammatory cytokines and inhibitory FcγRs (FcγRIIb) that counteract the signals mediated by activating FcγRs [18]. The most important FcγR on monocytes are medium-to-low affinity FcγRIIa, FcγRIIIa, and FcγRIIb that preferentially bind IgG ICs [18]. The role of FcγR-IC interactions in the pathology of COVID-19 was indirectly supported by findings that critically ill COVID-19 patients produced a unique serological signature, anti-SARS CoV-2 IgGs with afucosylated glycans that when incorporated into IC, trigger the FcγRIIIa activating receptor leading to production of proinflammatory cytokines in monocytes and macrophages [19]. Although higher concentrations of CoV-2 spike-IgG ICs were recently reported in the sera from severe COVID-19 [20], whether spike-IgG IC alone can activate monocytes or other ligands contribute to induction of inflammatory and pyrogenic mediators in monocytes have not been fully elucidated.

The immunopathology in severe COVID-19 is often accompanied by coagulation activation, thrombosis, and disseminated intravascular coagulation (DIC) [21]. The most frequently described report related to COVID-19 coagulopathy, is an increase in plasma D-dimer (DD) levels. DD is a small protein of 180  kDa that accumulates in the blood following degradation of stabilized fibrin polymer (fibrin crosslinked with factor XIII) by plasmin [22]. Importantly, many reports showed that elevated levels of DD are associated with disease severity [9,23,24]. Circulating proinflammatory cytokines IL-6 and IL-8, PGE2, and DD, are independently associated with a greater risk of COVID-19 disease severity and mortality; however, a link between these markers has not been investigated.

Here, we show that human monocytes exposed to IC of SARS CoV-2 pseudovirus with plasma from COVID-19 patients but not from healthy subjects, produce PGE2 and IL-6, IL-8, and IL-1β cytokines only in the presence of soluble DD. The quantities of cytokines and PGE2 produced by monocytes correlated with levels of SARS CoV-2 S protein binding antibodies but not with neutralizing antibody titers or DD in patients’ plasma. These data provide evidence for the role of DD as a potential link between blood coagulation and inflammation and suggest that targeted antagonism of this pathway would improve outcomes in patients with severe SARS CoV-2 infection.

## Results

### D-dimer induced production of PGE2 and proinflammatory cytokines IL-6 and IL-8 in human monocytes is augmented by immune complexes (ICs) containing SARS CoV-2 recombinant S protein (rS) and anti-spike IgG

Early studies on the inflammatory effect of the Fibrin Degradation Product (FDP), D-dimer (DD), showed that DD induced secretion of proinflammatory cytokines in promonocytic cells lines and in primary monocytes [25,26]. From the beginning of the COVID-19 pandemic, DD emerged as a prognostic factor of severe COVID-19: DD levels above 0.23 μg/ml were detected in two thirds of hospitalized COVID-19 patients and DD above 2 μg/ml was shown to associate with in-hospital mortality [9,27,28]. In addition, increased levels of proinflammatory cytokines and pyrogenic substances including IL-6, IL-8, IL-1β, and PGE2 were detected in patients with severe COVID-19 where they were proposed to play a role in recruitment of leukocytes including monocytes and neutrophils to the site of high viral replication in the lungs and subsequently contribute to ARDS [8–10]. To explore the link between increased levels of DD, PGE2, and proinflammatory cytokines in severe COVID-19, we sought to determine the DD dose response in human monocytes isolated by counter-flow centrifugal elutriation (elutriated monocytes)

**Fig 1:**
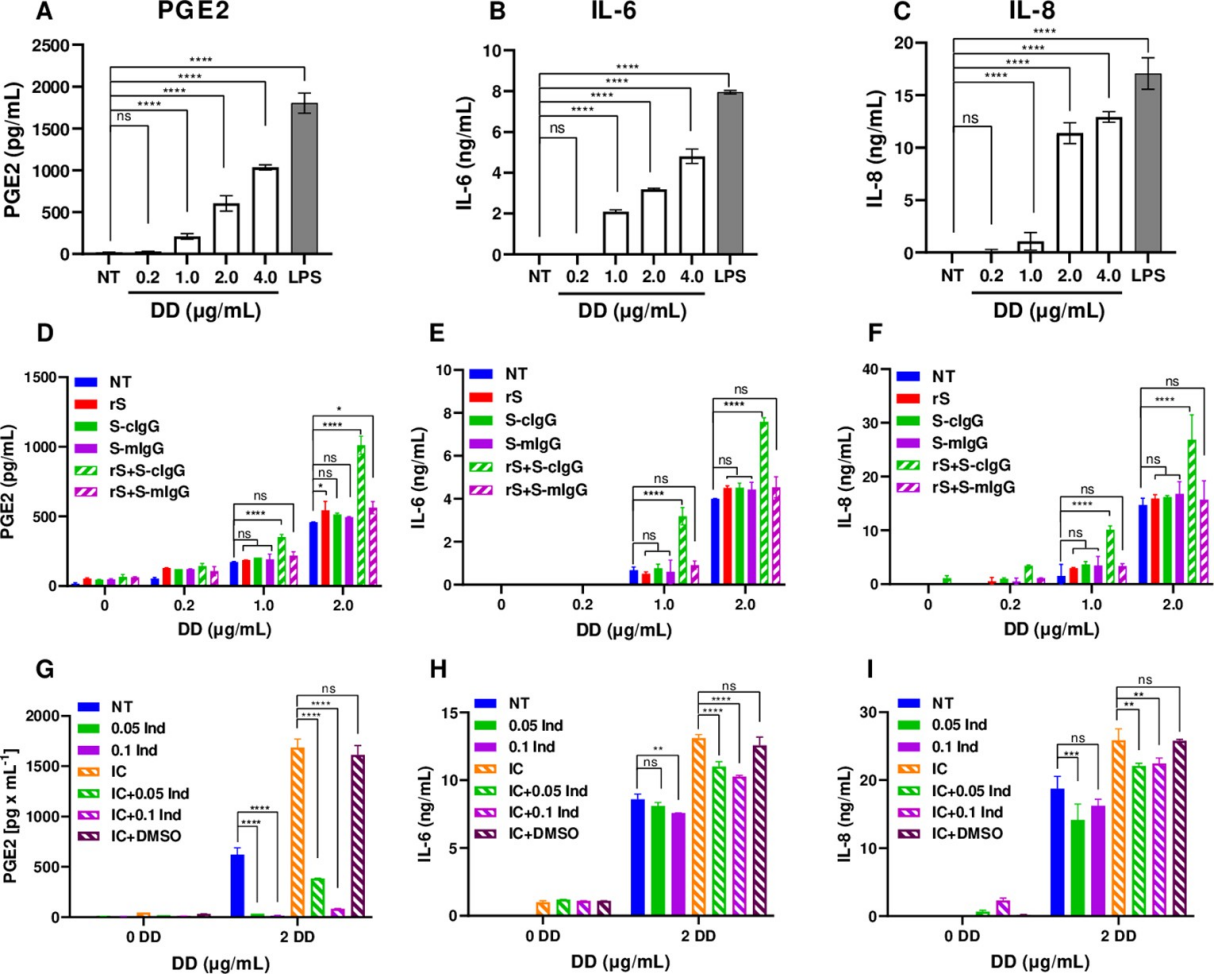
Production of PGE2, IL-6, and IL-8 in monocytes activated with DD in the presence of IC of SARS-CoV-2 rS protein with anti-CoV-2 spike MAb. Production of PGE2, IL-6, and IL-8 cytokines in monocytes activated with DD alone (A-C), with DD and IC (D-F), or with DD and IC in the presence of inhibitor of COX-2, indomethacin (G-I). Monocytes were left not treated (NT, A-I) or were incubated with DD or with LPS at 100 ng/ml (A-C) or with DD in the presence of rS, S-cIgG, S-mIgG, or IC of rS+S-cIgG or rS+S-mIgG (D-F) overnight. Monocytes were incubated with DD alone or with DD and rS+S-cIgG IC (IC) alone or in the presence of indomethacin (Ind at 0.05 or 0.1 μM) or with DMSO (G-I). Cell culture supernatants were collected and PGE2 (A, D, and G), IL-6 (B, E, and H) and IL-8 (C, F, and I) concentrations were determined by FRET assay (A, B) and by ELISA (B, C, E, F). Mean PGE2 and IL-6, and IL-8 concentrations ± SD calculated for triplicate wells are shown for a representative of three experiments performed with cells from individual donors. ****P ≤ 0.0001, by one-way ANOVA with Sidak’s multiple comparison test (A-C), and *P ≤ 0.05, ****P ≤ 0.0001 by two-way ANOVA using Tukey’s multiple comparison test (D-I).

Lyophilized DD protein from human plasma (from Abcam) was reconstituted and diluted according to the concentration indicated on the label. The quantity of native DD in the lyophilized protein was determined using antibodies specific for native DD in ELISA and was found to be about 5-fold lower than specified in the label (S1 Table). The amounts of DD used in all cell cultures of monocytes were adjusted based on the ELISA data (S1 Table). Monocytes were cultured overnight not treated (NT) or were incubated with DD at 0.2–4.0 μg/ml or with LPS at 100 ng/ml as positive control. Cell culture supernatants were assayed for PGE2 by Fluorescence Resonance Energy Transfer (FRET) assay (Fig 1A). A dose dependent increase in PGE2 production was observed between 1.0 and 4.0 μg/ml of DD. The maximum amounts of PGE2 induced by DD at 4.0 μg/ml was lower than PGE2 in monocytes treated with the TLR4-agonist LPS at 100 ng/ml (Fig 1A).

The same cell culture supernatants were also assayed for the presence of IL-6 and IL-8 cytokines by ELISA. Similar to PGE2, increasing levels of IL-6 were detected in monocytes treated with 1.0, 2.0, or 4.0 μg/ml of DD (Fig 1B). In the case of IL-8, the levels of IL-8 in monocytes treated with 2.0 or 4.0 μg/ml of DD were in the same range as in LPS-treated monocytes (Fig 1C). These results confirmed that DD at physiologically relevant concentrations of 1.0 to 4.0 μg/ml induced PGE2, IL-6, and IL-8 cytokines in human monocytes in a dose-dependent manner.

Recently, elevated levels of spike-IgG immune complexes (ICs) were reported in sera from patients with severe COVID-19 disease [20]. To explore whether spike-IgG ICs can induce PGE2 and inflammatory cytokines in monocytes, we initially tested spike-specific chimeric monoclonal antibody (MAb) combining the mouse variable region (S-cIgG) specific for CoV-2 spike with the human IgG-Fc domain. A fully mouse MAb against SARS CoV-2 spike protein (S-mIgG) was used as a control. Immune complexes (IC) were generated by incubating recombinant SARS-CoV-2 spike protein (rS) with the chimeric anti-spike IgG (rS+S-cIgG) or with the fully mouse anti-spike IgG (rS+S-mIgG). Monocytes were left not treated (NT) or were incubated with rS protein alone, S-cIgG alone, S-mIgG alone, or treated with ICs: rS+S-cIgG or rS+S-mIgG alone or in the presence of DD at 0.2, 1.0, or 2.0 μg/ml overnight. Cell culture supernatants were assayed for PGE2, IL-6, and IL-8 (Fig 1D, 1E and 1F). No increase in production of PGE2, IL-6, or IL-8 in monocytes incubated with rS protein in the presence of DD over DD alone was detected (Fig 1D, 1E and 1F). However, IC containing rS protein coated with chimeric spike specific MAb (rS+S-cIgG), but not fully mouse MAb (rS+S-mIgG), induced a two-fold increase in PGE2 (Fig 1D), IL-6 (Fig 1E), and IL-8 (Fig 1F) in the presence of DD at 1.0 or at 2.0 μg/ml compared with DD alone. A COX-2 inhibitor, indomethacin, added to monocytes at 0.05 or 0.1 μM, reduced production of PGE_2_ in monocytes activated with DD and rS+S-cIgG IC by 76% or 95% at low and high dose, respectively (Fig 1G). In contrast, IL-6 and IL-8 cytokines were only modestly reduced by indomethacin, 12% and 15–23% reduction for low and high dose, respectively, suggesting that their production in monocytes activated with DD and IC is primarily independent of PGE_2_ (Fig 1H and 1I). No PGE2, IL-6, or IL-8 cytokines were detected in cell cultures of monocytes incubated with rS+S-cIgG ICs alone or in the presence of 0.2 μg/ml of DD (Fig 1D, 1E and 1F, respectively). These data suggested that DD in combination with spike/anti-spike (human Fc) immune complexes enhanced production of PGE2 and proinflammatory cytokines in monocytes.

### Blocking of FcγRIIa and of CD18 integrin reduces production of PGE2 and cytokines in human monocytes activated with rS+S-cIgG IC in the presence of DD

**Fig 2:**
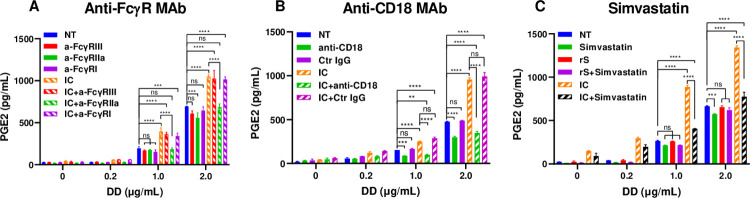
Inhibition of FcγRIIa and of CD18 reduced PGE2 production in human monocytes activated with spike-IgG IC in the presence of DD. Monocytes were left not treated (NT, in A-C) or were treated with anti-FcγR MAbs alone: anti-FcγRIII, anti-FcγRIIa, or anti-FcγRI MAbs (A), or with anti-CD18 MAbs or control IgG (Ctr IgG) alone (B) or with IC rS+S-cIgG alone (A and B), or with IC in the presence of anti-FcγR MAb: IC+anti-FcγRIII, IC+anti-FcγRIIa, or IC+anti-FcγRI (A) or with IC in the presence of anti-CD18 MAb or control IgG, IC+anti-CD18 and IC+Ctr IgG, respectively (B) and were incubated alone or in the presence of DD. (C) Monocytes were incubated with Simvastatin alone (8 nM), with rS protein alone or with rS+S-cIgG IC alone (IC) or with rS or IC in the presence of simvastatin, rS+Simvastatin and IC+Simvastatin, respectively, alone or in the presence of DD. Cell culture supernatants were collected and PGE2 concentrations were determined by FRET assay (A-C). Mean PGE2 concentrations ± SD calculated for triplicate wells are shown for a representative of three experiments performed with cells from individual donors. *P ≤ 0.01, **P ≤ 0.001, ***P ≤ 0.0001, by two-way ANOVA using Tukey’s multiple comparison test.

IC can be recognized by Fcγ receptors (FcγRs) expressed on monocytes including FcγRI, FcγRIIa, and FcγRIII. To determine which FcγRs are involved in activation of PGE2 production in monocytes exposed to rS+S-cIgG IC, we blocked different FcγRs with specific antibodies during the overnight incubation of monocytes with IC. Monocytes were left not treated (NT) or were pretreated with anti-FcγRI (CD64), anti-FcγRIIa (CD32), or anti-FcγRIII (CD16) MAbs for one hour and were subsequently incubated overnight with rS+S-cIgG IC alone or in the presence of DD at 0.2, 1.0, or 2.0 μg/ml (Fig 2A). Anti-FcγRIIa but not anti-FcγRIII or anti-FcγRI MAbs reduced PGE2 production in monocytes co-treated with rS+S-cIgG with DD to the levels observed in monocytes treated with DD alone (Fig 2A). Anti-FcγR MAbs alone did not alter significantly PGE2 production in monocytes not treated or treated with DD at 0.2–2.0 μg/ml.

Two β2 integrins, αMβ2 (CD11b/CD18, Mac-1) and αXβ2 (CD11c/CD18) are the main fibrinogen receptors expressed on monocytes, neutrophils, and macrophages. The recognition sites for β2 integrins have been mapped to the γ-module of the D-domain of fibrinogen [29]. To explore whether β2 integrin (CD18) plays a role in the production of PGE2 by monocytes activated with IC in the presence of DD, anti-CD18 Mab were added to monocytes (Fig 2B). Anti-CD18 MAb, but not isotype control IgG, significantly reduced PGE2 production in monocytes activated with IC in the presence of DD and in monocytes treated with DD in the absence of IC (Fig 2B). To further confirm the role of β2 integrin (CD18) in up-regulation of PGE2 in DD-treated monocytes, we used Simvastatin, a cholesterol lowering and anti-inflammatory drug that has been found to reduce CD18 expression in human monocytes in hypercholesterolemic patients and to reduce CD18 expression in TNFα-activated PBMC in vitro (Fig 2C) [30]. Pretreatment of monocytes with Simvastatin reduced PGE2 production by two-fold in monocytes co-activated with DD and spike IC (rS+S-cIgG) (Fig 2C). Together, these data demonstrate that FcγRIIa and β2 integrin (CD18) are the likely receptors responsible for induction of PGE2 in monocytes treated with anti-spike-IgG IC and DD, respectively.

### Role of calcium, MAPK, and Syk protein tyrosine kinase in production of PGE2 and cytokines in monocytes activated with anti-spike-IgG IC in the presence of DD

To dissect the signaling pathways involved in upregulation of pyrogenic and inflammatory mediators in monocytes activated with DD and spike IC (rS+S-cIgG), we measured production of PGE2, IL-6, and IL-8 in monocytes activated with DD alone or with DD combined with IC in the presence of inhibitor of calcium signaling, inhibitor of Mitogen-Activated Protein Kinase (MAPK) pathway or tyrosine-protein kinases.

**Fig 3:**
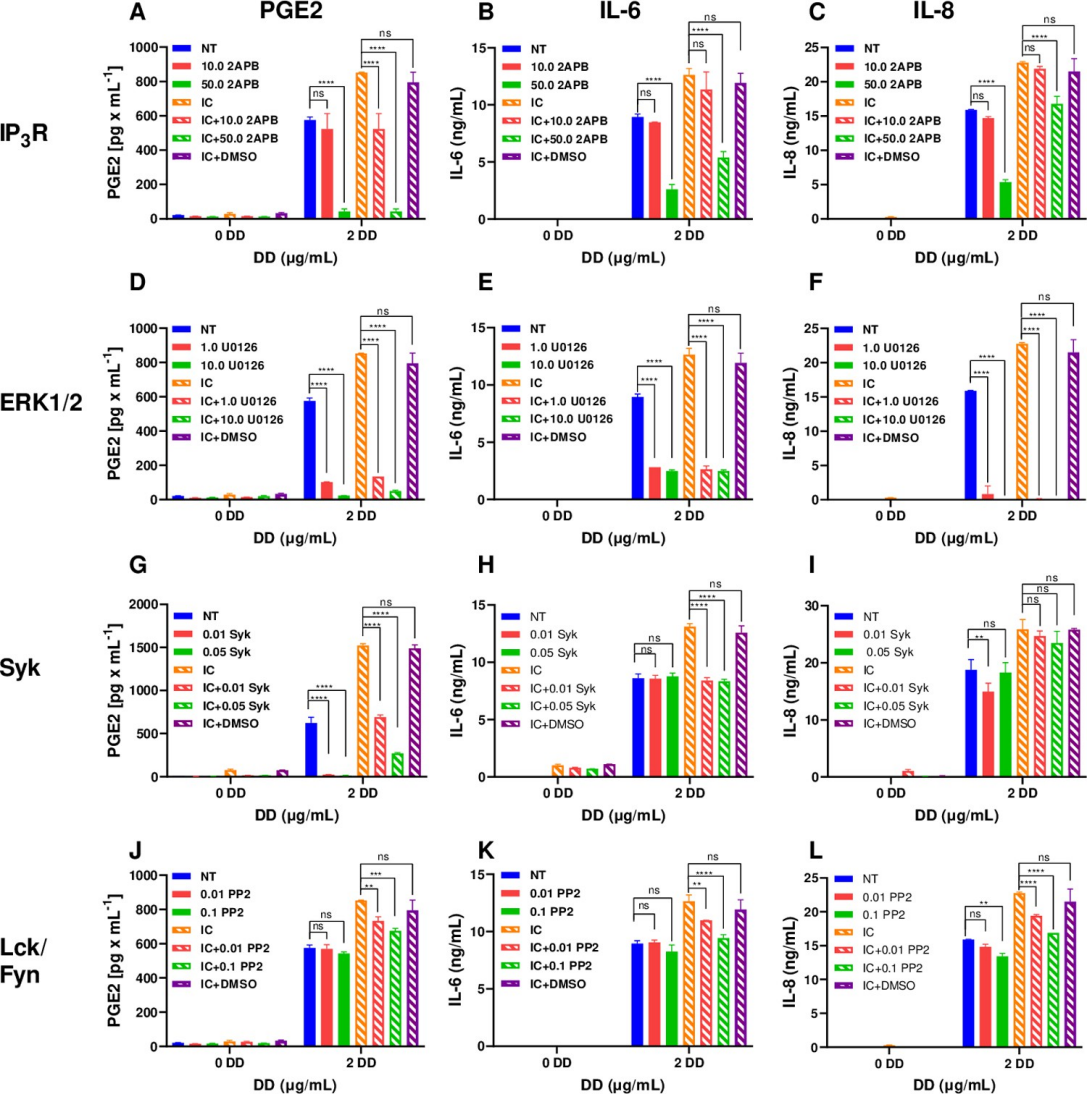
Inhibition of cytosolic [Ca2+], MAPK, and Syk protein tyrosine kinase differentially reduced production of PGE2, IL-6, and IL-8 in human monocytes activated with spike-IgG IC in the presence of DD. Monocytes were left not treated (NT in A-L) or were treated with inhibitors: inhibitor of InsP_3_R (2-ABP at 10 or 50 μM, A-C), inhibitor of ERK1/2 (U0126 at 1.0 or 10 μM, D-F), inhibitor of Syk kinase (PRT062608 at 0.01 or 0.05 μM, G-I), inhibitor of Lck/Fyn (PP2 at 0.01 or 0.1 μM, J-L), or with DMSO in control (A-L) and were incubated with rS+S-cIgG IC (IC) with DD at 2 μg/ml or with DD alone. Cell culture supernatants were collected and PGE2 (A, D, G, and J), IL-6 (B, E, H, and K), and IL-8 (C, F, I, and L) concentrations were determined by FRET assay (A, D, G, and J) and by ELISA (B, E, H, and K for IL-6 and C, F, I, and L for IL-8). Mean PGE2 and IL-6 and IL-8 concentrations ± SD calculated for triplicate wells are shown for a representative of three experiments performed with cells from individual donors. **P ≤ 0.001, ***P ≤ 0.0001, by two-way ANOVA using Tukey’s multiple comparison test.

The inhibitor of inositol 1,4,5-triphosphate receptor (InsP_3_R), 2-ABP, that prevents release of calcium from ER to the cytosol, reduced production of PGE2, IL-6, and IL-8 cytokines in monocytes activated with DD in the absence of IC by 92%, 68%, and 64%, respectively, when used at 50 μM concentration (Fig 3A–3C). Interestingly, 2-ABP completely inhibited PGE2 production and only partially reduced IL-6 (57%) and IL-8 (25%) in DD+IC-activated monocytes (Fig 3A–3C).

The MAPK signaling pathway is responsible for activation of transcription factors such as NF-κB (p50/p65) that coordinate the induction of genes encoding inflammatory mediators. The inhibitor of extracellular signal-regulated 1/2 (ERK1/2) MAP kinase, U0126, blocked production of PGE2 and of IL-6 and IL-8 in monocytes activated with DD alone or with DD combined with IC, suggesting that ERK1/2 plays a primary role in DD+IC-induced activation of both, PGE2 and inflammatory cytokines (Fig 3D–3F).

A non-receptor spleen tyrosine kinase (Syk), that plays a crucial role in signaling from FcR, was also shown to transmit signaling from other cell surface receptors including C-type lectins and integrins [31]. A Syk-specific inhibitor, PRT062608 (PP2), blocked production of PGE2 in DD-activated monocytes and partially reduced PGE2 in monocytes activated with DD+IC (55% and 82% reduction at low and high dose of PP2, respectively) (Fig 3G). At the same time, PP2 reduced production of IL-6 by 50% in DD+IC activated monocytes but not in DD alone activated monocytes and did not or only minimally reduced IL-8 (Fig 3H and 3I). Inhibitor of Lck tyrosine kinase had minimal effect on PGE2/IL-6/IL-8 production in monocytes activated with DD or with DD+IC (Fig 3G–3L).

Together, these data showed that DD or DD in combination with spike-IgG IC triggers multiple signaling pathways in monocytes that include cytosolic calcium, MAPK, and Syk protein tyrosine kinase, and that the relative contribution of each signaling pathway may vary between PGE2, IL-6, and IL-8 induction.

### IC of SARS CoV-2 pseudovirus with anti-spike chimeric MAbs induced PGE2, IL-6, and IL-8 cytokines in monocytes in the presence of DD

**Fig 4:**
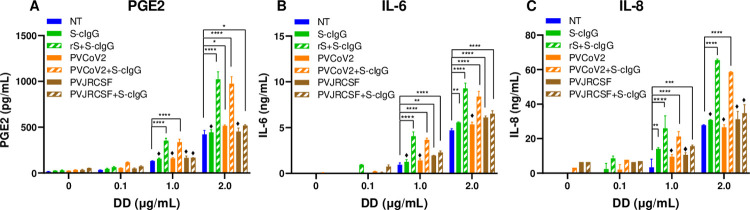
DD enhanced production of PGE2, IL-6, and IL-8 in monocytes activated with IC of SARS-CoV-2 pseudovirus coated with anti-CoV-2 spike MAb. (A-C) Monocytes were left not treated (NT) or were incubated with S-cIgG, with IC rS+S-cIgG, with PVCoV-2, with IC PVCoV-2+S-cIgG, with PVJRCSF, or with IC PVJRCSF+S-cIgG alone or in the presence of DD. Cell culture supernatants were collected and PGE2 (A), IL-6 (B) and IL-8 (C) concentrations were determined by FRET assay (A) and by ELISA (B, C). Mean PGE2 and IL-6, and IL-8 concentrations ± SD calculated for triplicate wells are shown for a representative of three experiments performed with cells from individual donors. *P ≤ 0.03, **P ≤ 0.01, ***P ≤ 0.001, ***P ≤ 0.0001, by two-way ANOVA using Tukey’s multiple comparison test; ♦, no significance (ns) compared with NT.

We next replaced recombinant spike protein with SARS-CoV-2 pseudovirus bearing full-length S glycoprotein (PVCoV-2) to form IC [32]. Pseudovirus expressing HIV-1 envelope protein from the JR-CSF isolate (PVJRCSF) was used as a negative control [33]. To that end, IC were generated by mixing MAb S-cIgG with 2 × 10^6^ RLU/mL of PVCoV-2 (PVCoV-2+S-cIgG) or with PVJRCSF (PVJRCSF+S-cIgG). IC (rS+S-cIgG) that was used in the previous experiments, was used as positive control (Fig 4). Monocytes were left not treated (NT) or were incubated with S-cIgG alone, with rS protein alone, with PVCoV-2 or PVJRCSF pseudoviruses alone, or were incubated with DD in the presence of ICs: rS+S-cIgG, PVCoV-2+S-cIgG, or PVJRCSF+S-cIgG overnight. Cell culture supernatants were assayed for PGE2, IL-6, and IL-8 (Fig 4A, 4B and 4C). IC containing PVCoV-2+S-cIgG but not IC of PVJRCSF+S-cIgG induced an increase in PGE2 production comparable to that induced by IC of rS-S-cIgG in monocytes co-incubated with DD at 1.0 or at 2.0 μg/ml over DD treatment alone. Similar patterns were observed when cell culture supernatants were assayed for IL-6 and IL-8 (Fig 4B and 4C). Treatment of monocytes with pseudoviruses PVCoV-2 or PVJRCSF in the presence of DD at 1.0 or at 2.0 μg/ml did not increase PGE2, IL-6, or IL-8 cytokines compared with DD treatment alone (Fig 4A, 4B and 4C).

### PGE2, IL-6, and IL-8 were induced in monocytes by IC of SARS CoV-2 pseudovirus with plasma from COVID-19 patients in the presence of DD

**Fig 5:**
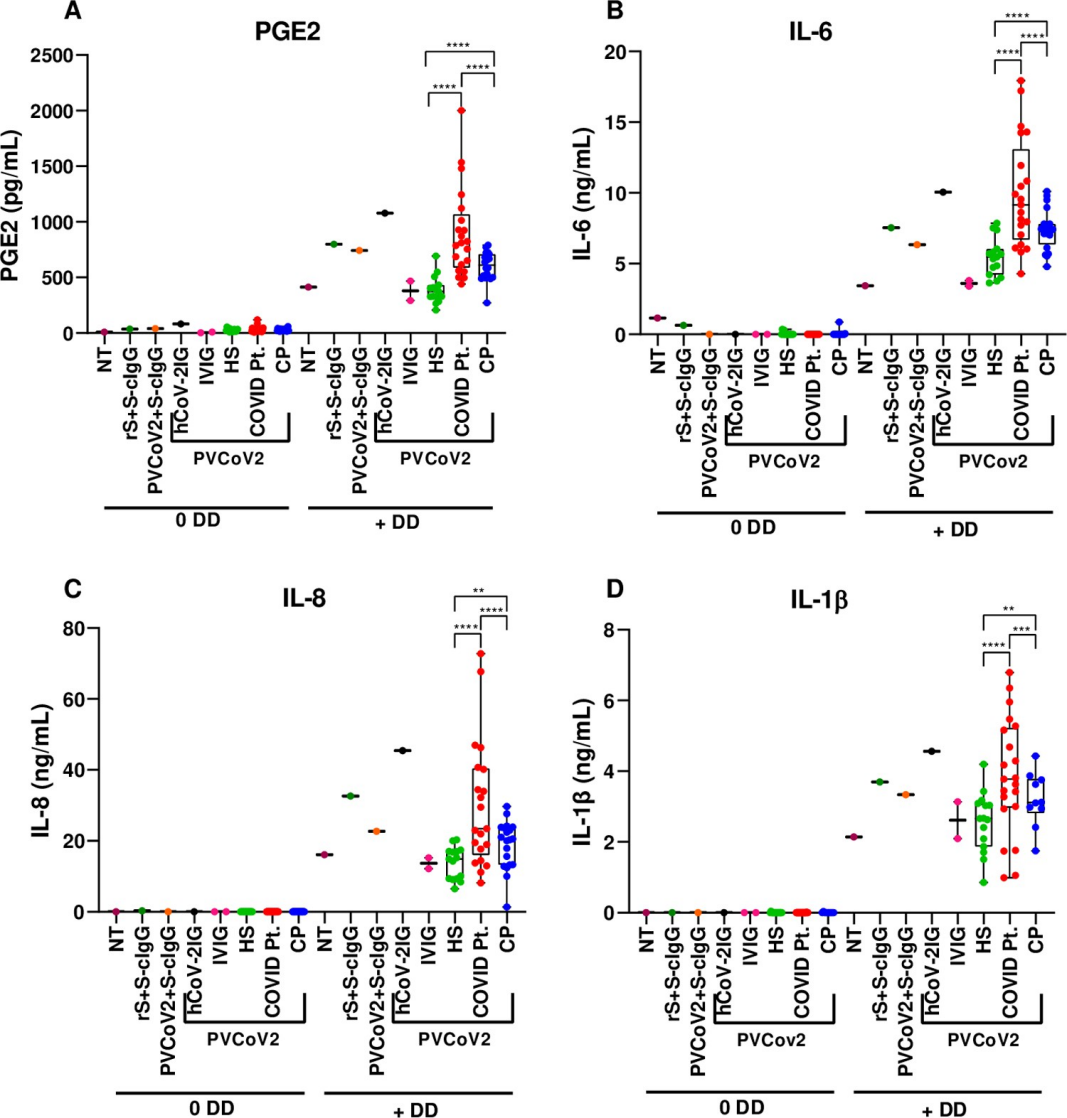
IC of SARS-CoV-2 pseudovirus with plasma from COVID-19 patients but not from healthy subjects induced PGE2, IL-6, IL-8, and IL-1β in monocytes in the presence of DD. Monocytes were left not treated (NT) or were incubated with rS+S-cIgG IC, with PVCoV-2+S-cIgG IC, or with IC of PVCoV-2 coated with hCoV-2IG (N = 1), IVIG (N-2), healthy subjects’ plasma (HS, N = 15), COVID-19 patients’ plasma (COVID Pt., N = 21), or convalescent plasma (CP, N = 20) alone (0 DD) or with 2 μg/ml of DD (+ DD) (A-D). Cell culture supernatants were collected and PGE2 (A), IL-6 (B), IL-8 (C), and IL-1β (D) concentrations were determined by FRET assay (A) and by ELISA (B-D). Mean PGE2 and IL-6, IL-8, and IL-1β concentrations ± SD calculated for triplicate wells are shown for a representative of two experiments performed with cells from individual donors. **P < 0.003, ***P < 0.001, ****P < 0.0001, by Kruskal-Wallis test with Dunn’s post-test comparison for individual groups.

Thus far we demonstrated increased PGE2/proinflammatory cytokine production using IC containing a chimeric MAb with human IgG Fc. It was important to determine whether immune complexes that may be formed in patients with severe form of COVID-19, activate monocytes alone or together with DD. In the next set of experiments, ICs were prepared by coating SARS CoV-2 pseudovirus with plasma samples from hospitalized COVID-19 patients (COVID Pt., N = 21), from healthy subjects (HS, N = 15), and from convalescent patients who recovered from COVID-19 (CP, N = 20), all diluted at 1:50. Additionally, hyperimmune IgG (100 mg/ml) (IVIG product) prepared from COVID-19 convalescent plasma (hCOV-2IG, N = 1) or from healthy uninfected subjects (IVIG, N = 2) were evaluated at 1:500 final dilution (Fig 5 and S2 Table). The neutralizing activity of all plasma samples was measured using a pseudovirion neutralization assay (PsVNA) (S2 Table) [34]. IC rS+S-cIgG and IC PVCoV-2+S-cIgG were used as positive controls for PGE2 and cytokine production. Monocytes were treated with IC of CoV-2 pseudovirus with plasma from all five sources in the absence or presence of DD at 2.0 μg/ml overnight and cell culture supernatants were assayed for PGE2, IL-6, IL-8, and IL-1β. IC containing plasma from healthy individuals or pre-pandemic IVIG did not augment the PGE2/IL-6/IL-8/IL-β levels in monocytes treated with DD compared with DD treatment alone (Fig 5A, 5B, 5C and 5D). In positive control cultures, a two-fold increase in PGE2, IL-6, IL-8, and IL-β was observed in monocytes treated with IC rS+S-cIgG or IC PVCoV-2+S-cIgG in the presence of DD compared with DD treatment alone, in agreement with the previous experiments. Importantly, IC of PVCoV-2 mixed with plasma from hospitalized COVID-19 patients (COVID Pt.) induced significantly higher levels of PGE2, IL-6, IL-8, and IL-β in DD-treated monocytes compared with DD treatment alone (Fig 5A, 5B, 5C and 5D). On the other hand, IC containing convalescent plasma (CP) induced only modest increase in PGE2 and cytokine production in DD-treated monocytes over DD alone (Fig 5A, 5B, 5C and 5D). IC formed with hCoV-2IG, manufactured from pre-screened CP with high antibody titers, also augmented the PGE2 and cytokine responses in DD-treated monocytes.

### The increase in PGE2 in human monocytes activated with IC in the presence of DD correlated with the levels of SARS CoV-2 spike binding antibodies

**Fig 6:**
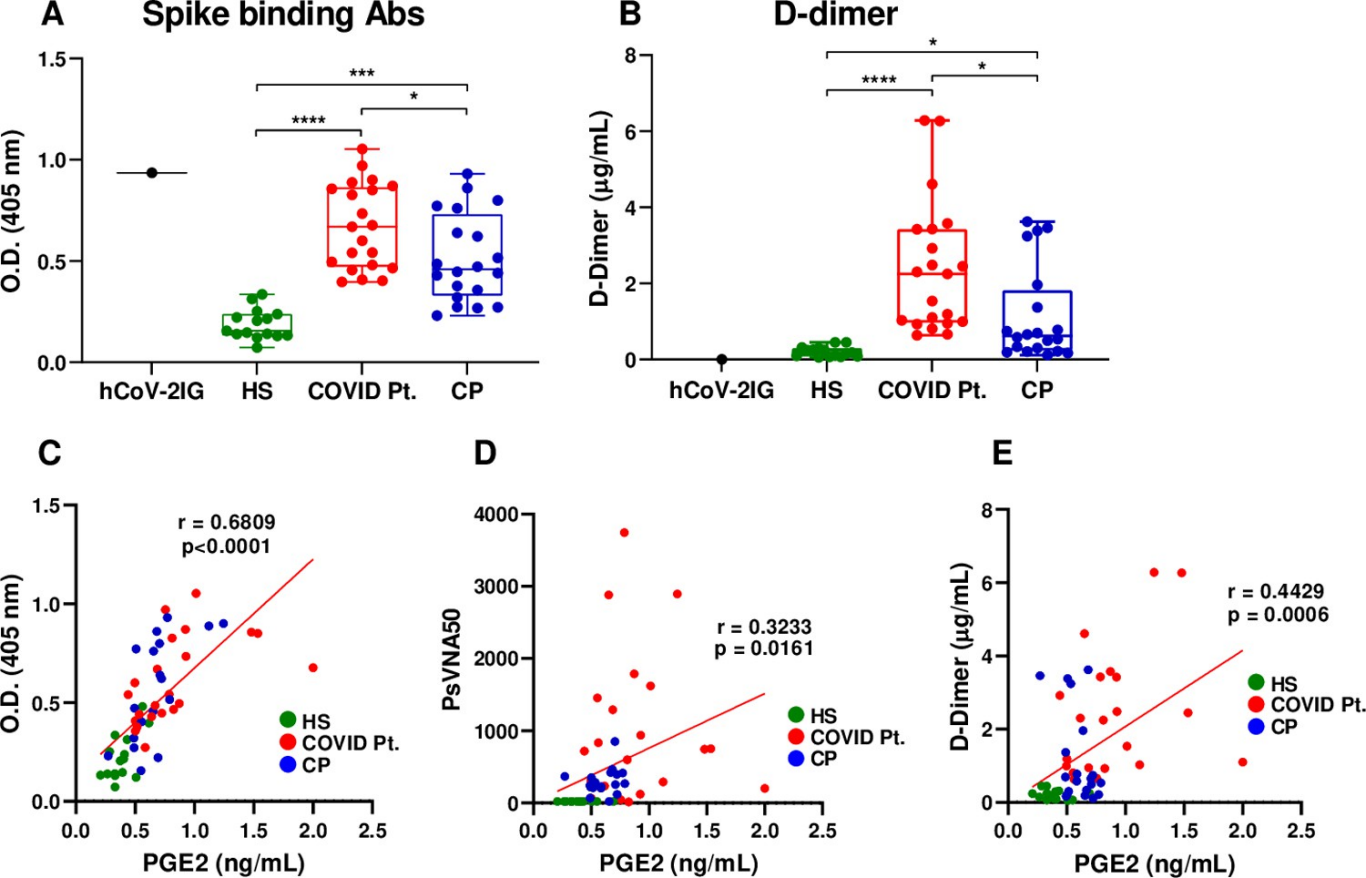
Strong correlation between PGE2 produced in monocyte cell culture and levels of anti-CoV-2 spike binding antibodies in human plasma. Quantities of SARS CoV-2 spike binding antibodies (A) and of DD (B) in samples of hCoV-2IG (N = 1), HS (N = 15), COVID Pt. (N = 21), and CP (N = 20) were measured by ELISA. Data is shown as mean antibody concentrations (O.D. at 405 nm) ± SD (A) and as mean DD concentrations ± SD (B). *P < 0.05, ***P < 0.005, ****P < 0.0001, by one-way ANOVA test followed by Dunn’s multiple comparison test. Correlation between the quantity of CoV-2 spike binding antibody (C) or of antibody neutralizing titers (PVsVNA50) (D) in plasma samples and quantity of PGE2 in monocyte cell culture supernatants or between PGE2 in monocyte cell culture supernatants and quantities of DD in human plasma (E). Pearson’s correlation coefficient (r) was calculated for plasma samples from healthy subjects, COVID-19 patients, and convalescent plasma; hCoV-2IG (a highly concentrated purified commercial IgG product) was not included in the analysis (C, D, and E).

To gain insight into the mechanism underlying the PGE2 production in healthy monocytes by IC of PVCoV-2 pseudovirion with plasma from COVID-19 patients, we analyzed the impact of three variables: quantity of anti-spike protein binding antibodies, SARS CoV-2 antibody neutralizing titers, and quantity of DD in plasma (Fig 6 and S2 Table). SARS CoV-2 spike binding antibodies and DD concentrations were measured by ELISA in plasma samples described in the legend to Fig 5. Higher levels of antibodies against SARS CoV-2 spike protein were detected in plasma samples from hospitalized COVID19 Pt. compared with CP. Plasma from healthy subjects (HS) had no or minimal binding activity (Fig 6A). The levels of DD were highly variable between donors and were significantly higher for the plasma from hospitalized COVID-19 patients vs. convalescent plasma, while plasma from healthy donors (HS) contained minimal levels of DD (Fig 6B). No DD was detected in hCOV-2IG (Fig 6B).

The levels PGE2 amounts detected in cell cultures of monocytes activated with IC of CoV-2 pseudovirus with plasma samples showed a strong correlation with the levels of spike protein binding antibodies, R = 0.6809, p < 0.0001 (Fig 6C). In contrast, the correlation between PGE2 production and PsVNA50 values was much weaker, R = 0.3233 (Fig 6D). Similarly, a strong correlation was observed between spike binding antibodies and the levels of IL-6 and IL-8 cytokines produced by monocytes activated with spike-IgG ICs, R = 0.6632 and R = 0.6475, respectively (S1A and S1B Fig). A modest correlation was found between plasma DD and PGE2 production in monocytes, R = 0.4429 (Fig 6E). Therefore, we cannot exclude a contribution of plasma DD to the overall production of PGE2 and inflammatory cytokines in our system, which still required addition of exogenous DD at ≥ 1 μg/ml for optimal responses. The correlation analysis suggested that the anti-spike binding antibody levels were the main driver of the IC-augmented inflammatory response.

The present results suggest that production of pyrogenic and inflammatory mediators in human monocytes is controlled through engagement of two signaling pathways: a priming signal, triggered by DD above threshold concentration through CD18, which is amplified (2 to 5-fold) by signal 2 derived from spike antibody-bound virus containing immune complexes that are more readily formed in patients with severe form of the disease due to high SARS CoV-2 viral loads and high levels of anti-CoV-2 spike antibodies.

## Discussion

**Fig 7:**
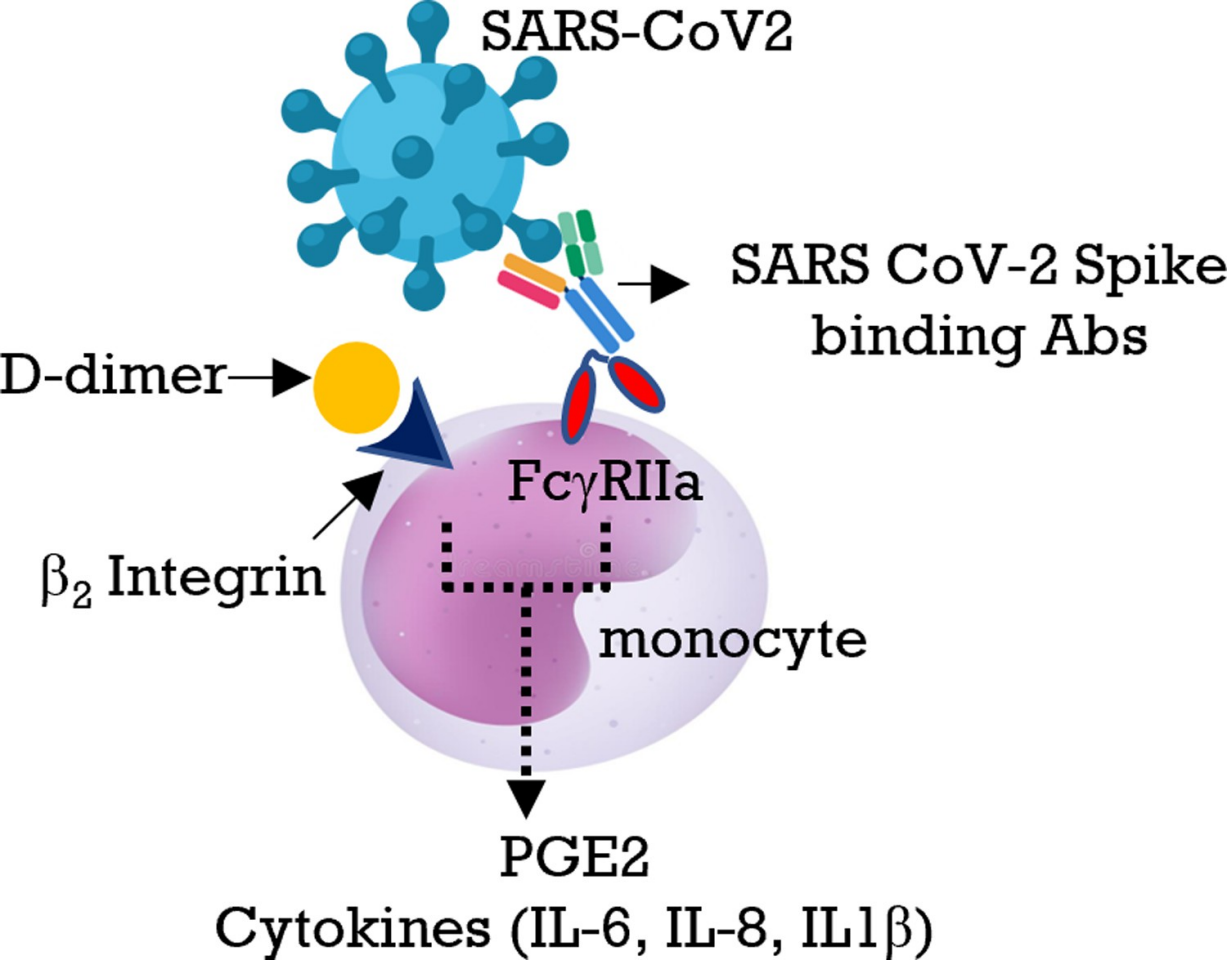
Model of PGE2 and cytokine production in monocytes activated with DD and IC. DD alone induces low levels of PGE2 and IL-6/IL-8 cytokines production in monocytes through β2 integrin. This priming signal is then amplified by signaling from FcγRIIa triggered by immune complexes of SARS CoV-2 viral particles coated with SARS CoV-2 spike binding antibodies leading increased production of PGE2, IL-6, and IL-8 by monocytes.

Respiratory failure or acute respiratory distress syndrome (ARDS) is the most common cause of death in COVID-19. Although the underlying mechanism of ARDS is not completely understood, it has been attributed to the excessive numbers of leukocytes recruited to the lungs of infected individuals together with a systemic cytokine storm, and COVID-19 associated coagulopathy. Multiple studies of risk factors associated with severe disease have shown that coagulation activation strongly correlates with increased levels of DD, a fibrinogen degradation product. In the present study, we explore the contribution of DD to the excessive inflammation in severe COVID-19. The results show that DD plays a crucial role in the induction of PGE2 and of inflammatory cytokines (IL-6 IL-8, IL-1β) in primary human monocytes, which is further increased by immune complexes containing virions coated with anti-spike antibodies (Fig 7). The combination of proinflammatory signals can lead to recruitment of inflammatory monocytes and other cells (i. e. neutrophils) to the site of high viral replication in the lungs culminating in ARDS.

Our data agree with the study that reported correlation between elevated levels of PGE2 in the blood and COVID-19 disease severity [11]. At the same time, recent ex vivo study showed that progression to severe COVID-19 was associated with reduced levels of COX-2 (a critical enzyme in PGE2 biosynthesis) in monocytes upon in vitro activation with LPS and suggested that COX-2 may play a positive role in protection from COVID-19 disease severity [35]. However, the immune response of monocytes from patients with severe disease to LPS ex vivo may be compromised by preceding treatments that these patients received in the hospital settings. Importantly, in our study, PGE2 production was measured in monocytes in response to DD and IC containing SARS CoV-2 pseudovirions coated with anti-spike antibodies that were found at elevated levels in patients with severe form of the disease. LPS may be not physiologically relevant agonist in these settings.

The potential impact of immune complexes on pathogenicity in COVID-19 was proposed [36] based on histopathology findings in the lung tissues that revealed features associated with IC mediated vasculitis including infiltration of monocytes and lymphocytes within and around blood vessels, vascular wall thickening, and focal hemorrhage [37,38]. In support of this notion, increased levels of CoV-2 spike-IgG ICs in the sera of COVID-19 patients was recently shown to correlate with disease severity [20]. Our study agrees with recent reports that demonstrated that IC of SARS CoV-2 virions with purified anti-spike IgG or with sera from patients with severe disease triggered increased production of proinflammatory cytokines (IL-6, TNF-α, and IL-1β) in monocytes [39] and in M2 macrophages [40]. The ability of spike-IgG to induce inflammatory mediators reported in these studies was attributed to the specific feature: spike-IgG from critically ill patients transiently exhibited lower fucosylation of the antibody Fc molecule that was previously suggested to play a major role in determining the affinity of IgGs to FcγR [41]. The requirement for priming by DD observed in our study was not reported. However, the levels of DD were not measured in the previous studies. We did not have sufficient volumes to conduct fucosylation analysis on the purified IgG antibodies from patient plasma. The immune complex mediated inflammatory signal in monocytes in our study was transmitted through FcγRIIa and not FcγRIII that was the primary FcγR for afucosylated IgG1 [39,40]. In our experiments, blocking of CD18 (integrin β chain 2) with anti-CD18 antibodies or with the chemical inhibitor, simvastatin, reduced PGE2 production in monocytes activated with DD confirming the role of β2 integrin in transmitting priming signal.

Previous studies have shown that activation of leukocytes and macrophages by FcγR-dependent mechanism can be controlled by signals from other surface receptors. Production of proinflammatory cytokines in IC-activated macrophage was strongly enhanced by Pam3CSK4 and LPS that activate TLR2 and TLR4 innate receptors, respectively [42]. Moreover, IC-induced Antibody Dependent Cytotoxicity (ADCC), recruitment, and migration of neutrophils required concurrent activation of αMβ2 integrin [43–45]. In agreement with these observations, our studies showed that a cross talk between FcγRIIa activated by spike-IgG IC and β2 integrin triggered by DD had an additive impact on the production of PGE2 and proinflammatory cytokines in monocytes.

Downstream signaling in monocytes activated with DD and IC was also investigated in our study. Experiments using multiple inhibitors showed that DD and IC utilize common as well unique signaling pathways: ERK1/2 MAP kinase was responsible for upregulation of PGE2, IL-6, and IL-8 suggesting that ERK1/2 plays indispensable role in activation of pyrogenic and pro-inflammatory mediators in monocytes activated with DD and IC. Cytosolic [Ca2+] was required for production of PGE2, IL-6, and IL-8 in monocytes activated with DD alone in agreement with our earlier observations that signaling from αMβ2 integrin increases cytosolic [Ca2+] in monocytes [46]. Interestingly, in monocytes activated with DD in the presence of IC, the contribution of calcium signaling varied as inhibitor of IP_3_R completely blocked PGE2 but not IL-6 or IL-8 production suggesting that production of pro-inflammatory cytokines was partially [Ca2+] independent. Although Syk kinase is considered the major kinase that transmits signaling from FcR, in our system, inhibition of Syk blocked or reduced PGE2 and IL-6, but not IL-8 in monocytes activated with DD and IC. These data suggested that production of IL-8 in DD+IC-activated monocytes is Syk independent and might be driven primarily by ERK1/2.

Studies of the immune response in COVID-19 patients have demonstrated that SARS CoV-2 induces a robust immune response with early appearance of antibodies targeting the nucleocapsid (N) and the spike protein of SARS CoV-2 virions followed by virus neutralizing antibodies. Although presence of antibodies is indicative of protection from infection, paradoxically, high titers of neutralizing antibodies were reported to correlate with disease severity [47–49]. Several mechanisms have been suggested to explain how higher humoral responses can promote disease progression including development of Antibody Dependent Enhanced (ADE) disease, a condition where pathogen-specific antibodies promote viral entry into FcR expressing cells and that have been shown for other CoV including SARS-CoV and MERS-CoV. A more recent study showed that direct ADE is not involved in severe forms of COVID-19 experienced by the patients [50]. In our experiments, the levels of PGE2 and proinflammatory cytokines produced by monocytes activated with IC in the presence of DD correlated well with the levels of total spike binding IgG antibodies and not with the individual neutralizing antibodies. Thus, our data provide a potential mechanism that may explain how high titers of SARS CoV-2 specific non-neutralizing antibodies may contribute to disease pathogenesis [51].

All plasma samples used in the study were also tested for the presence of endogenous DD. Although no PGE2 and cytokine levels were noted in the absence of exogenous DD (Fig 4), we cannot exclude the possibility that internal DD, present in plasma samples from severe COVID-19 patients, contributes to the IC-mediated activation of monocytes. IC formed with plasma from convalescent patients (plasma from individuals following COVID-19 resolution) induced significantly lower levels of PGE2 and cytokines compared with plasma from COVID-19 patients, however the highly concentrated hCoVIG product was more potent. Our findings suggest that usage of such products for treatments of patients with severe form of the disease should be carried out with caution.

Progressive coagulation activation and concurrent activation of fibrinolysis within the lungs has been shown to coincide with increased levels of DD in the blood in patients with severe disease [52,53]. The rise in DD in circulation was suggested to occur following transfer of fibrin, present in the alveoli and lung parenchyma because of the lung injury, from the local lung to the blood stream [54]. Therefore, it is possible to speculate that circulating monocytes that are actively recruited to the infected lungs might be activated locally by DD transiently located in the lungs.

## Conclusion

Early in the COVID-19 pandemic, conflicting data was reported regarding the effect of anticoagulant (AC) therapy on thrombosis and survival in critically ill patients infected with SARS CoV-2. Some reports concluded that intermediate and high dose of AC therapy were beneficial in prevention of incidents of in-hospital death and other studies did not support this conclusion [55–57]. This pressing issue was recently addressed in four large randomized controlled clinical trials, REMAP-CAP, ACTIV-4, ATTACC, and HEP-COVID (NCT 02735707, 04505774, 04372589, and 04401293, respectively) that compared the effect of therapeutic-intensity and prophylactic-intensity AC in hospitalized patients with COVID-19 [58–60]. Despite some differences in the trial design, these studies showed that therapeutic AC with low molecular weight or unfractionated heparin was associated with improved outcomes in hospitalized patients with COVID-19 who were not critically ill or in ICU setting. At the same time, patients, who were critically ill and/or were in the ICU, did not benefit from therapeutic AC. Thus findings described in our ex vivo experiments are indirectly supported by clinical observations and suggest a mechanism for the reported benefit of early administration of AC therapies prior to disease exacerbation and cytokine storm.

## Materials and methods

### Ethics statement

The study protocol was approved by Center for Biologics Evaluation and Research (CBER), Food and Drug Administration (FDA) and performed under study protocol #CBER-2020-04-09 on de-identified plasma donations obtained from COVID-19 patients at the Adventist HealthCare White Oak Medical Center (Silver Spring, MD) and Shady Grove Medical Center (Rockville, MD). This study complied with all relevant ethical regulations for work with human participants, and informed consent was obtained. All adults hospitalized with COVID-19 disease were eligible without any specific selection criteria. Samples were collected from patients who provided written informed consent to participate in the study. All assays performed fell within the permissible usages in the original informed consent.

### Plasma samples

Heat inactivated plasma samples were obtained from Washington Adventist Medical Health Care and Shady Grove Adventist Hospital through Quest Diagnostics. The plasma samples were collected on the day of hospital admission (acute samples, N = 21) or 4–6 weeks after discharge (convalescent samples, N = 20). All samples were deidentified. Initially, no patient information was provided, and all the immune analyses were conducted blindly. Subsequent patient information was provided and is presented in Source Data for the patients evaluated in the current study. This study was approved Food and Drug Administration’s Research Involving Human Subjects Committee (FDA-RIHSC) (RIHSC #2020-04-02).

Two hCoV-2IG batches prepared from 250–400 COVID-19 CP donors per lot were obtained from commercial companies under MTA. The plasma units used in the manufacturing of the hCoV-2IG batches were collected in 2020 prior to emergence of variants of concern in the U.S.

The intravenous immunoglobulin (IVIG) batch was produced from plasma collected prior to August 2019 (from >10,000 donors). The final product is sterile-filtered IgG (purity—95%) and formulated at 100 mg/mL.

Antibody preparations were evaluated by SARS-CoV-2 pseudovirus neutralization assay (PsVNA) using WA-1 strain as previously described [34].

### Preparation of immune complexes (IC)

Immune complexes were prepared using the following reagents: SARS-CoV-2 recombinant protein (rS) (SinoBiological, Wayne, PA, Cat 40589-V08B1), SARS CoV-2 pseudovirus bearing full length S glycoprotein (PVCoV-2), pseudovirus expressing HIV-1 envelope protein from the JR-CSF isolate (PVJRCSF) [32,33], anti-SARS-CoV-2 Spike MAb (S-cIgG), a chimeric IgG combining the constant domains of the human IgG1 with mouse variable regions (S-cIgG) (the variable region was obtained from a mouse immunized with recombinant SARS CoV-2 Spike RBD Protein) (SinoBiological, Cat 40150-D003), and SARS CoV-2 spike mouse MAb (IgG1) (SinoBiological Cat 40591-MM43).

ICs were prepared by incubating 8 μg/mL of S-cIgG with SARS CoV-2 rS protein at 20 μg/mL or with either PVCoV-2 or PVJRCSF at 2 × 10^6^ RLU/mL. IC with human plasma were prepared by incubating PVCoV-2 at 2 × 10^6^ RLU/mL with plasma samples from hospitalized COVID-19 patients (COVID Pt., N = 21) or from healthy subjects (HS, N = 15) or with convalescent plasma (CP, N = 20), (all at 1:50), or with hyperimmune IgG (IVIG product) (100 mg/ml) (hCOV-2IG, N = 1) or from healthy uninfected subject (IVIG, N = 2) (at 1:500) for 2 hr at 37°C and 5% CO_2_ with gentle rotation.

### Cells and cell treatment

Human monocytes isolated from healthy donors using counter-flow centrifugal elutriation (elutriated monocytes) were obtained from the Department of Transfusion at the National Institutes of Health (NIH, Bethesda, MD). Elutriated monocytes were 100% CD14+ as verified by Flow Cytometry.

Monocytes were cultured at 15 × 10^6^ cells/mL with ICs and DD (Abcam, Waltham, WA, Cat ab281292 Lot GR282157-22) in a complete RPMI1640 medium with 10% FBS and supplements in 96-well plates at 37°C and 5% CO_2_ overnight. In some experiments, monocytes were preincubated for 30 min before adding IC and DD with the following MAbs at 4 μg/mL: anti-human FcγRI (CD64) (GeneTex, Irvine, CA, Cat GTX76484, Clone 10.1), anti-human FcγRII (CD32) (GeneTex, Cat GTX14572, Clone IV.3), anti-human FcγRIII (CD16) (Biolegend, San Diego, CA, Cat 302002, Clone 3G8), anti-human CD18 (Invitrogen, Waltham, MA, Cat MA1810, clone TS1/18), or mouse IgG1 mAb isotype control (Cell Signaling, Manvers, MA, Cat 5415S, clone G3A1). In some experiments, monocytes were pre-incubated with simvastatin (MK733, Selleckchem, Huston, TX, Cat S1792) at 8 nM or with indomethacin (0.05 and 0.1 μM, MilliporeSigma, Burlington, MA, Cat I7378), 2-ABP (10 and 50 μM, MilliporeSigma, Cat 100065), U0126 (1.0 and 10 μM, MilliporeSigma, Cat 19–147), PRT062608 (0.01 and 0.5 μM, Selleckchem, Cat I7378), or PP2 (0.01 and 0.1 μM, Bio-Techne Corporation, Pittsburgh, PA, Cat 1407) for 1 hr prior to treatment with DD and IC. Cell viability was controlled in monocytes and no toxicity was detected in treated cells in all conditions.

### Measurements of PGE2 by fluorescence resonance energy transfer (FRET) assay and of IL-6, IL-8, and IL-1b cytokines, and of DD by enzyme-linked immunosorbent assay (ELISA)

Cell culture supernatants from monocytes were collected and assayed for PGE2 by fluorescence resonance energy transfer (FRET) assay using PGE2 Homogeneous Time-Resolved Fluorescence assay (HTRF) Kit (Cisbio Bioassays Bedford, MA) and FLUOstar plate reader (BMG Labtech, Cary, NC) as previously described [61]. PGE2 concentration from culture media were calculated using four-parameter logistic fit using Omega Data Analysis software application (BMG Labtech, version 5.50 R4). The detectable PGE2 range was from 10 to 5000 pg/mL. IL-6, IL-8, and IL-1β cytokines were measured in the monocyte cell culture supernatants using human Quantikine enzyme-linked immunosorbent assay (ELISA) kits (R&D systems, Minneapolis, MN). DD in human plasma, in hCoV-2IG, and in IVIG was measured by colorimetric quantitative sandwich ELISA kit (Human DD ELISA Kit, Abcam). All plates were read by Synergy2 Multi-Mode plate reader (BioTek Instruments, Winooski, VT).

### Measurement of anti-SARS CoV-2 spike binding antibody in human plasma by ELISA

SARS-CoV-2 Spike rS protein at 1μg/ml in carbonate-bicarbonate ELISA coating buffer (pH 9.6) was coated on MaxiSorp 96-well plate (Nunc, Rochester, NY) overnight at 4°C. Plates were blocked with 5% milk in PBS-Tween buffer (0.5% Tween 20 v/v) for 1 hour at 37°C. Human plasma samples (1:100) were added in 50 μL volumes to each well and were incubated overnight at 4°C. Plates were incubated with 50 μl of Anti-human (H+L) HRP conjugated antibody (1:5000) for 1 hr at room temperature followed by HRP substrate for 15 ~ 20 min at 37°C, both from SeraCare (Gaithersburg, MD). The reaction was stopped using 1% SDS stopping solution. ELISA plates were read at 405 nm optical density using Synergy2 plate reader.

### Statistical analysis

Statistical analysis was performed using GraphPad Prism software, version 8.4.3. Data were analyzed with unpaired two-tailed Student’s t test and significance of differences was determined by one-way ANOVA with Sidak’s multiple comparisons test or by one-way ANOVA followed by Dunn’s post hoc analysis, or by two-way ANOVA using Tukey’s multiple comparison test. Kruskal-Wallis test with Dunn’s post-hoc analysis was employed for comparison between individual groups. Pearson’s coefficient and p-value were calculated to evaluate correlation between PGE2 levels and spike binding antibodies, neutralizing antibody titers, or DD quantities in plasma. P values of ≤ 0.05 were considered statistically significant. Sample sizes for each experimental condition and details of the statistical analyses are provided in figure legends.

## Supporting information

S1 Fig Production of IL-6 and IL-8 in monocytes activated with IC and D-dimer correlated with the quantity of plasma CoV-2 spike binding antibodies (O.D.) in plasma. (PDF)

S1 Table Quantity of native D-dimer in reconstituted lyophilized D-dimer protein. (PDF)

S2 Table Source of human plasma, ICU vs non-ICU status of COVID-19 patients, and SARS CoV-2 antibody neutralizing titers in plasma samples used in the study. (DOCX)

S1 Data Data file for S1 Fig. (XLSX)

S2 Data Data file for Figs 1, 2, 3, 4, 5 and 6. (XLSX)
