# Supplementary material for: D-dimer and CoV-2 spike-immune complexes contribute to the production of PGE2 and proinflammatory cytokines in monocytes
Source: PLoS Pathog. 2022 Apr 6;18(4):e1010468. doi: 10.1371/journal.ppat.1010468 (PMC9015149; doi:10.1371/journal.ppat.1010468)
Supplement: S1 Fig — (PDF) [file ppat.1010468.s001.pdf]

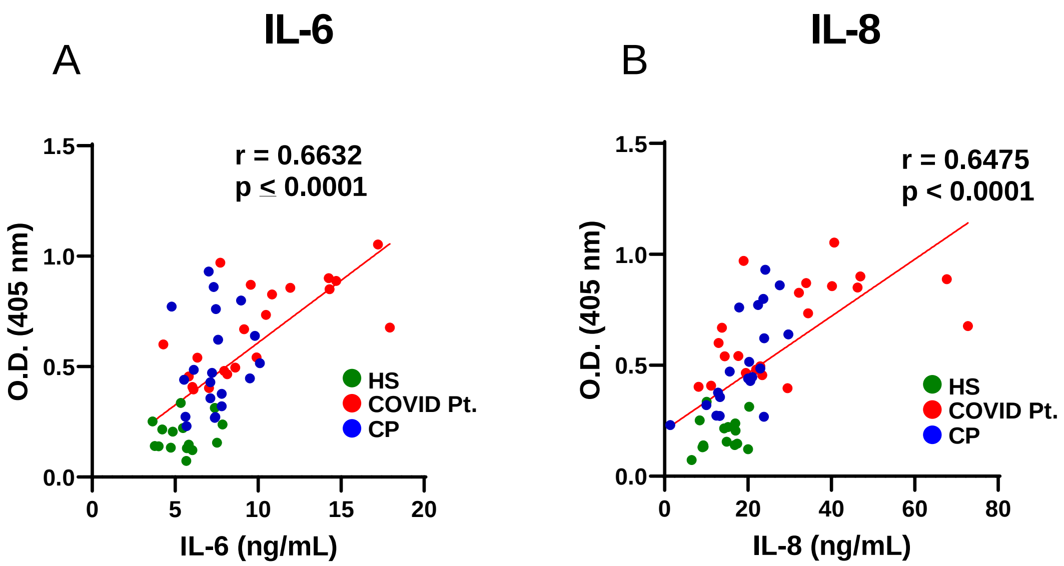


**S1 Fig. Production of IL-6 and IL-8 in monocytes activated with IC and D-dimer correlated with the quantity of plasma CoV-2 spike binding antibodies (O.D.) in plasma.** Correlation between quantity of IL-6 (A) and IL-8 (B) cytokines in monocyte cell culture supernatants and quantity of SARS CoV-2 spike binding antibodies (O.D.) in plasma. Pearson's correlation coefficient (r) was calculated for plasma samples from healthy subjects, COVID-19 patients, and convalescent plasma.
