# Supplementary material for: D-dimer and CoV-2 spike-immune complexes contribute to the production of PGE2 and proinflammatory cytokines in monocytes
Source: PLoS Pathog. 2022 Apr 6;18(4):e1010468. doi: 10.1371/journal.ppat.1010468 (PMC9015149; doi:10.1371/journal.ppat.1010468)
Supplement: S1 Table — (PDF) [file ppat.1010468.s002.pdf]

S1 Table Quantity of native D-dimer in reconstituted lyophilized D-dimer protein

D-dimer concentration	D-dimer (µg/ml)					
Label[1]	0.5	1.0	3.0	5.0	7.5	10.0
Measured[2]	0.08	0.17	0.55	0.9	1.36	1.82

[1] Quantity of D-dimer in dilutions prepared according to the concentration indicated on the label (Abcam, Cat ab281292, Lot GR282157-22)

[2] Concentration of native D-dimer as measured by Human D-Dimer ELISA Kit (Abcam, Cat ab260076).
