# Supplementary material for: D-dimer and CoV-2 spike-immune complexes contribute to the production of PGE2 and proinflammatory cytokines in monocytes
Source: PLoS Pathog. 2022 Apr 6;18(4):e1010468. doi: 10.1371/journal.ppat.1010468 (PMC9015149; doi:10.1371/journal.ppat.1010468)
Supplement: S2 Table — (DOCX) [file ppat.1010468.s003.docx]

S2 Table Source of human plasma, ICU vs non-ICU status of COVID-19 patients, and SARS CoV-2 antibody neutralizing titers in plasma samples used in the study

| Sample ID | Source | Neut. Titer^1^ | ICU Status |
| --- | --- | --- | --- |
| 1-1 | COVID-19 Patient | 1786.7 | ICU |
| 1-2 | COVID-19 Patient | 3743.6 | ICU |
| 1-3 | COVID-19 Patient | 64.3 | Non-ICU |
| 1-4 | COVID-19 Patient | 745.3 | Non-ICU |
| 1-5 | COVID-19 Patient | 201.6 | Non-ICU |
| 1-6 | COVID-19 Patient | 14.9 | Non-ICU |
| 1-7 | COVID-19 Patient | 939.7 | Non-ICU |
| 1-8 | COVID-19 Patient | 752.6 | Non-ICU |
| 1-9 | COVID-19 Patient | 718.4 | Non-ICU |
| 1-10 | COVID-19 Patient | 308.1 | Non-ICU |
| 7-1 | COVID-19 Patient | 1293 | Non-ICU |
| 7-2 | COVID-19 Patient | 235.8 | Non-ICU |
| 7-3 | COVID-19 Patient | 45.12 | Non-ICU |
| 7-4 | COVID-19 Patient | 833.8 | Non-ICU |
| 7-5 | COVID-19 Patient | 1623 | Non-ICU |
| 7-6 | COVID-19 Patient | 291.3 | Non-ICU |
| 7-7 | COVID-19 Patient | 121.2 | Non-ICU |
| 7-8 | COVID-19 Patient | 598.5 | Non-ICU |
| 7-9 | COVID-19 Patient | 2880 | Non-ICU |
| 7-10 | COVID-19 Patient | 2896 | Non-ICU |
| 7-11 | COVID-19 Patient | 1455 | Non-ICU |
| 2-1 | Convalescent Plasma | 119.6 | NA |
| 2-2 | Convalescent Plasma | <20 | NA |
| 2-3 | Convalescent Plasma | 67 | NA |
| 2-4 | Convalescent Plasma | 211.1 | NA |
| 2-5 | Convalescent Plasma | 268 | NA |
| 2-6 | Convalescent Plasma | 204.9 | NA |
| 2-7 | Convalescent Plasma | 369.2 | NA |
| 2-8 | Convalescent Plasma | 243.1 | NA |
| 2-9 | Convalescent Plasma | 852.7 | NA |
| 2-10 | Convalescent Plasma | 9141.1 | NA |
| 4-1 | Convalescent Plasma | 218.85 | NA |
| 4-2 | Convalescent Plasma | 350.5 | NA |
| 4-3 | Convalescent Plasma | 465.3 | NA |
| 4-4 | Convalescent Plasma | 416.1 | NA |
| 4-5 | Convalescent Plasma | 395.5 | NA |
| 4-6 | Convalescent Plasma | 253.9 | NA |
| 4-7 | Convalescent Plasma | 227.5 | NA |
| 4-8 | Convalescent Plasma | 226.2 | NA |
| 4-9 | Convalescent Plasma | 420.3 | NA |
| 4-10 | Convalescent Plasma | 285.8 | NA |
| 6-1 | Healthy Control plasma | <20 | NA |
| 6-2 | Healthy Control plasma | <20 | NA |
| 6-3 | Healthy Control plasma | <20 | NA |
| 6-4 | Healthy Control plasma | <20 | NA |
| 6-5 | Healthy Control plasma | <20 | NA |
| 6-6 | Healthy Control plasma | <20 | NA |
| 6-7 | Healthy Control plasma) | <20 | NA |
| 6-8 | Healthy Control plasma | <20 | NA |
| 6-9 | Healthy Control plasma | <20 | NA |
| 6-10 | Healthy Control plasma | <20 | NA |
| 3-1 | Healthy Control plasma | <20 | NA |
| 3-2 | Healthy Control plasma | <20 | NA |
| 3-3 | Healthy Control plasma | <20 | NA |
| 3-4 | Healthy Control plasma | <20 | NA |
| 3-5 | Healthy Control plasma | <20 | NA |
| 5-1 | hCoV-2IG | 22420 | NA |
| 5-2 | IVIG-1 | <20 | NA |
| 5-3 | IVIG-2 | <20 | NA |

^1^ The neutralizing activity of all plasma samples and of hCoV-2IG and IVIG was measured using pseudovirion neutralization assay (PsVNA) as previously described (Ref 34).

NA, Not available
